# Editorial: Dermatopathology and Associated Laboratory Investigations in the Study of Skin Disease

**DOI:** 10.3389/bjbs.2025.14810

**Published:** 2025-06-13

**Authors:** Guy Edward Orchard

**Affiliations:** St John’s Dermatopathology, Synnovis Analytics, St. Thomas’ Hospital, London, United Kingdom

**Keywords:** dermatopathology, mycology, fungus, gout, immunofluorescence

In this Special Issue for the *British Journal of Biomedical Science* we see a mixture of papers, some original articles, some case studies and some reviews. All of these articles seek to contribute to the understanding and advancement in knowledge acquisition into the subject of dermatopathology and associated laboratory-based investigations in the study of skin disease. Each paper in this Special Issue deals with particular aspects on laboratory-based investigations into a wide range of skin diseases dealing with both inflammatory and cancerous conditions. Each of these papers in turn discusses current practice and techniques and shows how these can help define and identify a myriad of differing conditions. This Special Issue shows the importance of clinic-pathological correlation which is so well entrenched in the discipline of dermatopathology.

Howell presents an overview of dermatopathology and the identification of fungal infections employing mycological techniques and explains how they are so closely linked. In the study of subcutaneous skin infectious fungal disease, the histological techniques evaluate a skin biopsy, which defines the inflammatory process and may highlight and direct mycological culture investigations into an infectious cause or foreign body type reaction. This demonstrates the importance of how these two disciplines interact to confirm a definite cause of a fungal infection’s states. Howell’s review goes on to explain the value of histopathological special stains such as periodic acid–Schiff (PAS) and Grocott and Gomori Methanamine Silver (GMS)which greatly improve the detection of fungal elements. Although, careful morphological examination of the tissue in combination with the relevant clinical history can indicate the type of fungal infection, it cannot indicate the causative species. Culture techniques are still vital to define fungal species and through this approach determine the course of treatment needed. Howell’s review also introduces the growing development of new molecular based diagnostic approaches to deliver faster diagnostic outcomes.

A case report and subsequent review by Orchard deals with a common inflammatory state involving Gout in a 73-year-old man, but, as in this case, unusually associated with AA amyloidosis. The paper explains the common dermatological findings of a cutaneous episode of a gout tophus and describes the classical macroscopic and dermatopathological features found. The identification of AA amyloid is described. AA amyloid is associated with chronic inflammatory changes and is often associated with deposits in the urine as well as tissue involvement. A review of only 16 previously reported cases in the literature of gout associated with AA is discussed investigating possible associations between the pathophysiology of these two processes. The paper also explores the evidence of the early use of anti-inflammatory drugs like colchicine before gout tophi formation as a mechanism to prevent the formation of AA amyloidosis.

Gabriel et al.’s original investigative paper evaluates the introduction of a new mordant based haematoxylin dye (Haematoxylin X) for use in clinical pathology. In this comparative study conventionally used alum based haematoxylins (Carazzi’s, Harris’ and Mayer’s) are compared to the new chromium based mordant Haematoxylin X on formalin fixed paraffin embedded tissue (FFPE) sections taken from a selection of normal and pathological skin tissue types, along with tissue sampled from a wide host of other organ sites. The new Haematoxylin X was also compared with use as a counterstain in both special stains and immunohistochemistry and in cases of frozen section Mohs procedures. In terms of sensitivity and specificity scores on FFPE Haematoxylin X was rated second behind Harris haematoxylin. However, assessments of frozen Mohs section staining demonstrated that Haematoxylin X scored the highest in terms of both sensitivity and specificity and was an effective counterstain in some special staining techniques and also as a counterstain haematoxylin for immunohistochemical techniques.

Following on from the use of conventional haematoxylin staining we have Mee’s review on the use of immunoflourescent (IMF) techniques in the study of autoimmune blistering diseases. In the review Mee highlights the fact that there is often significant overlap in clinical appearances in patients with cutaneous autoimmune states affecting the basement membrane zone (BMZ), with diagnosis often dependent on the identification and the detection and characterisation of specific autoantibodies. IMF provides the “gold standard” diagnostic approach in such scenario’s identifying either tissue bound autoantibodies investigated from biopsies taken from lesion and perilesional sites of blistered affected skin, termed direct IMF or from circulating antibodies found within the patient’s serum termed indirect IMF. Following on from this the characterisation of numerous antigenic targets in these group of diseases has involved the development of antigen specific tests most notably those based on enzyme-linked immunosorbent assays on serum from affected patients. Mee explains that by the use of all three of these approaches to the study of affected skin tissue and patient serum provides, evidence to define the autoimmune blistering disease process and thus establish the opitmal treatment regime to follow can be defined, as some of these conditions do also have an association and increased risk of malignancy.

Continuing on the theme of malignancy Salih et al.’s original paper on the use of a double labelling technique involving Prame and MelanA in cases of FFPE tissue sections in cutaneous slow Mohs tissue to assess cases of Lentigo Maligna (LM) and Lentigo Maligna Melanoma (LMM) highlights a novel and useful adjunct technique to assist in the evaluation of margin clearance in such cases. Morphological examination of surgical margin clearance is key to determining successful treatment in LM/LMM in slow Mohs cases. The use of preferentially expressed antigen in melanoma (Prame) to assist in such assessments is gaining in popularity. A total of 51 slow Mohs cases of LM/LMM were evaluated using the double labelling technique and compared to single labelling techniques. The results indicated that there was a high concordance between double labelling and single labelling techniques across the samples assessed. The Prame single labelling technique exhibited a sensitivity of 91.3% in slow Mohs cases with a 67.9% concordance in histologically confirmed positive margins. The study highlights the utility for the use of Prame immunohistochemistry and also Prame double labelling techniques as an adjunct to the assessment of LM/LMM in staged slow Mohs cases.

Finally, to finish off this special issue, Gabriel et al. have a narrative review of molecular, immunohistochemical and *in situ* techniques in dermatopathology. The review highlights the significant impact of skin disorders generally within the wider global population, highlighting how it can affect millions of individuals across diverse demographics. More recent advancements in molecular techniques have improved our understanding of the underlying mechanisms of skin disorders generally along with significant improvements in the application of molecular diagnostic testing in clinical practice. Immunohistochemistry and fluorescence *in situ* hybridisation have also played their part in determining protein expression patterns and in detecting chromosomal abnormalities, which in turn help characterise skin lesions in conjunction with the molecular data. This review explores the scope and range of these techniques, it highlights the combined approaches within dermatopathology, with a clear focus on cutaneous malignancies, autoimmune diseases, infectious diseases and neonatal screening approaches, which can be employed in both a diagnostic setting and also to aid in the improvement of patient treatment regimes. Gabriel et al explain the steady rise in molecular technology applications within the field of dermatopathology, but emphasises the importance of international collaboration to test the application of these technologies thoroughly before they become widely accepted in clinical use. This in conjunction with the requirement to comply with *in vitro* diagnostic regulations throughout Europe plus the constant need in investment in novel equipment which will assist in the evaluation of increasing numbers of molecular biomarkers remain key challenges for the future. Gabriel et al concludes by stating that molecular techniques represent a vital tool in the clinicopathological correlation of skin disease states generally and will enhance the information acquired from conventional light microscopic studies.

These six papers, covering a wide range of differing subject matter, technological advances and varied methodologies highlight the scope and importance of why dermatopathology is often key in the diagnosis of skin disease and also explain its relationship and importance to links with other specialised laboratory services that help define and characterise cutaneous disorders.

